# Antigenic and Pathogenic Characteristics of QX-Type Avian Infectious Bronchitis Virus Strains Isolated in Southwestern China

**DOI:** 10.3390/v11121154

**Published:** 2019-12-13

**Authors:** Shuyun Li, Lijing Du, Jing Xia, Jiteng Du, Guojin You, Yiping Wen, Xiaobo Huang, Qing Zhao, Xinfeng Han, Qigui Yan, Rui Wu, Min Cui, Sanjie Cao, Yong Huang

**Affiliations:** Key Laboratory of Animal Disease and Human Health of Sichuan Province, College of Veterinary Medicine, Sichuan Agricultural University, Huimin Road 211, Wenjiang, Chengdu, Sichuan 611130, China; lishuyun607@163.com (S.L.); dulijing1228@163.com (L.D.); xiajing1028@163.com (J.X.); djt0514@163.com (J.D.); 15998938536@163.com (G.Y.); yueliang5189@163.com (Y.W.); hxb010@126.com (X.H.); zhaoqinde@foxmail.com (Q.Z.); hanxinf@163.com (X.H.); yanqigui@126.com (Q.Y.); wurui1977@163.com (R.W.); cuimintracy@163.com (M.C.); csanjie@sicau.edu.cn (S.C.)

**Keywords:** infectious bronchitis virus, IBV, QX, antigenic, pathogenic

## Abstract

The QX-type avian infectious bronchitis virus (IBV) is still a prevalent genotype in Southwestern China. To analyze the antigenicity and pathogenicity characteristics of the dominant genotype strains (QX-type), S1 gene sequence analysis, virus cross-neutralization tests, and pathogenicity test of eight QX-type IBV isolates were conducted. Sequence analysis showed that the nucleotide homology between the eight strains was high, but distantly related to H120 and 4/91 vaccine strains. Cross-neutralization tests showed that all eight strains isolated from 2015 and 2017 belonged to the same serotype, but exhibited antigenic variations over time. The pathogenicity test of the five QX-type IBV isolates showed that only three strains, CK/CH/SC/DYW/16, CK/CH/SC/MS/17, and CK/CH/SC/GH/15, had a high mortality rate with strong respiratory and renal pathogenicity, whereas CK/CH/SC/PZ/17 and CK/CH/SC/DYYJ/17 caused only mild clinical symptoms and tissue lesions. Our results indicate that the prevalent QX-type IBVs displayed antigenic variations and pathogenicity difference. These findings may provide reference for research on the evolution of IBV and vaccine preparation of infectious bronchitis (IB).

## 1. Introduction

Infectious bronchitis (IB) is a highly contagious disease that causes significant economic losses to the poultry industry worldwide [1,2]. Furthermore, chickens infected with infectious bronchitis virus (IBV) also become susceptible to secondary infections with bacteria, or other pathogens, because of the tracheal cilia damage, and display a higher mortality rate [3]. IBV, an etiological agent of IB, is an enveloped virus with a single-stranded positive-sense non-segmented RNA genome of approximately 27.6 kb in length and belongs to the *Gammacoronavirus* genus [4]. Due to the incomplete proofreading mechanism of coronavirus RNA polymerase and gene recombination during viral replication, new genotypes and serotypes of IBV variant strains appear continuously [5,6]. An array of serotypes and strains of IBV infect chickens exist throughout the world. Therefore, continuous testing of pathogenicity and serotype determination of new isolates in regions and/or countries remains crucial for better epidemiological understanding and control of IB.

The spike 1 (S1) gene is highly variable among IBV strains and encodes epitopes, which can induce the production of specific neutralizing antibodies [7]. The partial or the full-length of the S gene has been used in the molecular characterization of IBV isolates. The antigenic relatedness and receptor binding with host cells are influenced by hypervariable regions (HVRs) in the S1 gene [8,9,10]; further, the S gene and 5a accessory gene are responsible for the attenuation of virulent IBV via a suitable reverse-genetic system [11]. Therefore, the differences in the genotypes, antigenicity, and pathogenicity existing among IBVs are mainly related to the S1 subunit of the IBV spike protein.

Since the early 1980s, IBV has been continuously isolated in China, and QX genotype has become the prevalent genotype [12,13,14]. Our previous study showed that isolates obtained from Southwestern China between 2008 and 2016 mainly belong to the QX genotype [12,15]. Other reports have shown that QX-type IBVs epidemic is a major problem in the poultry industry in Europe, Japan, Korea, Russia, Africa, and the Middle East [16,17,18]. However, even with the wide use of IBV vaccines, such as H120 and 4/91 for QX-type IBV infection, immune failure occurred frequently [4,19]. QX-like IBV has become a challenge to the prevention and control IBV. Therefore, it is crucial to gain a better understanding of the antigenicity and pathogenesis of QX-like IBV. Previously, comparative studies have been conducted to elucidate differential pathogenicity among two QX-like IBVs with 94.1% similarities of the S1 gene [20]. Very minor changes in the IBV genome can lead to differences in pathogenicity [21]; therefore, comparative studies involving more QX-type IBVs are required. The present study was conducted with the aim to elucidate the S1 gene differences of eight QX-type IBVs isolated from vaccinated chicken flocks in Southwestern China and describe its antigenicity and pathogenicity characteristics.

## 2. Materials and Methods

### 2.1. Viruses and Eggs

Eight QX-type IBVs, CK/CH/SC/GH/15, CK/CH/SC/MS/15, CK/CH/SC/DYW/16, CK/CH/SC/XSH/17, CK/CH/SC/PZ/17, CK/CH/SC/MS/17, CK/CH/SC/DYYJ/17, and Sczy3 (hereafter referred to as GH/15, MS/15, DYW/16, XSH/17, PZ/17, MS/17, DYYJ/17, and Sczy3, respectively), were included in the study. Of these isolates, the Sczy3 strain was identified as QX-type and SCZY3-serotype by a previous study (GenBank number: JF732903.1) [12]. The other seven field strains were isolated from clinical samples (trachea, lung, and kidney) of dead/diseased chickens from Southwestern China that displayed respiratory symptoms and/or nephritis during 2015–2017. Virus isolation was carried out by being inoculated into the allantoic cavity of 9-to-10-day-old specific-pathogen-free (SPF) chicken embryos with 0.2 mL of 10% tissue homogenates. The embryos were incubated at 37 °C and examined twice daily for their viability. The allantoic fluids wereharvestedafter36 h of incubation, and three blind passages were conducted. Detection methods of presence of IBV were determined based on the previous literature [12]. The 50% embryo infectious doses (EID_50_) were determined as previously described (Reed and Muench, 1938) [22]. SPF chicken embryos were obtained from the Beijing Merial Vital Laboratory Animal Technology Co., Ltd. (Beijing, China) and were hatched at our laboratory (Lab of Chicken Infectious Disease, College of Veterinary Medicine, Sichuan Agricultural University, Chengdu, China). The details of the seven field isolates are shown in Table 1.

### 2.2. Phylogenetic and Recombination Analysis of S1 Genes

Viral RNA was extracted from fresh allantoic-fluid-infected IBVs, using a TRIzol agent, as per the manufacture’s recommendation (Invitrogen, Carlsbad, CA, USA). Reverse transcription was performed, using a reverse-transcriptase (RT) kit (Takara Bio-Inc, Dalian, China), according to the manufacturer’s protocol. PCR amplification of IBV S1 gene sequencing was referred to in the previous study [12,15]. The nucleotide sequences of the S1 gene, including isolates and reference strains, were aligned by using the ClustalW method in MEGA version 7.0.14 [23]. The information of 18 references is shown in Table S1. The phylogenetic tree of the S1 gene created by a neighbor-joining tree method was drawn in MEGA version 7.0.14 [24]. Bootstrap values were determined from 1000 replicates. Recombination events of the potential recombinant strain were estimated, using the Recombination Detection Program (RDP 4.72) and SimPlot version 3.5.1 software, as previously reported [12].

### 2.3. Antigenic Analysis of Different QX-Type Strains

#### 2.3.1. Preparation of Chicken Antisera against IBVs

Chicken antisera were prepared against the eight strains. Briefly, fresh allantoic of 10^4^ EID_50_ IBVs were inactivated, using 0.1% formal, at 37 °C, for 18 h. The inactivated mixture was emulsified with an equal volume of complete Freund’s adjuvant (Sigma, Saint Louis, MO, USA). Following this, 0.1 mL of the mixture was used for subcutaneous injection in four-week-old SPF chickens (*n* = 3) as first immunization. Subsequently, the same amount of antigen was emulsified in Freund’s incomplete adjuvant (Sigma, Saint Louis, MO, USA) for the following two booster injections (at two-week intervals). A serum sample of each immunized chicken was collected 14 days after immunization and inactivated at 56 °C for 30 min.

#### 2.3.2. Adaption of IBVs in Chicken Embryo Kidney (CEK) Cells

CEK cells were prepared and cultured as previously described [15]. Primary CEK cells were prepared from the kidneys of SPF chicken embryos at 18–20 days old and maintained in Dulbecco’s Modified Eagle Medium (DMEM; Gibco, Grand Island, NY, USA) supplemented with 10% fetal bovine serum (FBS; Zhejiang Tian-hang Biological Technology Stock Co., Ltd., Zhejiang, China) [25]. Those IBVs were propagated in primary CEK cells cultured in DMEM supplemented with 2% FBS at 37 °C under 5% CO_2_. The supernatant was harvested at 40 h post-inoculation and passaged blindly in CEK cells until a characteristic cytopathic effect (CPE), such as syncytia, was observed. The presence of IBV in the supernatant of the CEK culture was verified by RT-PCR, as previously reported [15]. Determination of the TCID_50_ of CEK-adapted IBVs in CEK cells was conducted as per the method reported by Reed and Muench (1938)

#### 2.3.3. Virus Cross-Neutralization Tests

The antigenic relatedness (R) of those eight strains was determined via double-direction viral cross-neutralization (VN) tests with constant viral titers and diluted serum in CEK cells [12]. Briefly, sera were serially diluted two-fold with sterile phosphate-buffered saline (PBS), and then equal volumes of 100 TCID_50_ of the CEK-adapted strains were added. The mixtures were then incubated at 37 °C for 1 h. Virus–serum mixtures were used to infect CEK cell cultures in 24-well plates (6 wells for each dilution) and incubated for 72 h. Following this, 50% end-point neutralizing titers were calculated, using the method described by Reed and Muench (1938). A negative serum sample was always included in the analysis.

#### 2.3.4. Antigenic Relatedness Analysis

The cross-reactivity R values were calculated according to a previously reported method, representing the relationship between antigens: R ≥ 0.70 proves antigenic identity, 0.70 > R ≥ 0.33 proves antigenic relatedness (minor subtype difference), and 0.33 > R ≥ 0.11 proves loose relatedness (major subtype difference), whereas R < 0.11 indicates no relatedness at all (serotype difference) [26,27]. Antigenic cartography was also performed by using the program AntigenMap (http://sysbio.cvm.msstate.edu/AntigenMap), which uses matrix completion multidimensional scaling to map microneutralization titers in two dimensions [28]. Viruses with high antigenic relevance clustered closely on the map, while viruses with low antigenic relevance stayed far away from each other. Detailed settings were set as follows: Low Reactor Threshold: 20; Normalization Method: N1; Temporal Model: Normal; Rank: 2; Number of Iterations: 2000.

### 2.4. Primer Design and Quantitative Real-Time RT-PCR Validation (RT-qPCR)

#### 2.4.1. Primer Design

One pair of specific primers (QX-F: AGCGGTAGTCTGAATCTGTTAAAG; QX-R:GAAGAACAAACTCATCGACATCC) was designed to amplify only the sequence of QX-type genotype IBV strains in quantitative real-time RT-PCR (RT-qPCR) detection of viral loads. One pair of primers (SF: CAGTAGCAAGTATCCTCTA; SR: GCTTTGGCAATTACAACAGTC) was designed for the amplification of standard plasmids. Primers were designed by using Oligo 7 and synthesized by Sanggong Biotechnology (Sanggong, Shanghai, China).

#### 2.4.2. Standard Plasmid

Viral RNA was extracted from fresh allantoic fluid, using TRIzol (Invitrogen, Carlsbad, CA, USA) according to the manual instructions. Viral cDNA was transcribed by using the reverse-transcriptase (RT) kit (Takara Bio-Inc, Dalian, China), following the manufacturer’s instructions. The fragments of IBV strains were amplified by using SF and SR by PCR reaction. The amplicons were then inserted into pMD-19T cloning vector (Takara Bio-Inc, Dalian, China) and transformed into *Escherichia coli* TOP10 competent cells (Takara Bio-Inc, Dalian, China). The recombinant plasmid DNA was extracted, and its copy numbers were calculated according to a previous report [29].

#### 2.4.3. RT-qPCR Assay

The RT-qPCR was performed by using the SYBR Premix Ex Taq TM II (Takara Bio-Inc, Dalian, China). The 20 μL reaction mix consisted of 10 μL of SYBR^®^ Premix Ex Taq^TM^II (TliRNaseH Plus, Takara), 0.8 μL of each specific primer (QX-F and QX-R: 10 pmol/μL), 1 μL of standard plasmid, and 7.4 μL of RNase-free ddH_2_O. The optimum thermal cycling parameters were as follows: 95 °C for 30 s, and 40 cycles of 95 °C for 10 s, 54 °C for 15 s, and 72 °C for 20 s. The dissolution curve was generated by the default parameters at the end of the cycle. Under optimum thermal cycling parameters, the standard curves of RT-qPCR were established by linear regression analysis of the threshold cycle (Ct) value (*y* axis) versus the log of the initial copy number presented in each sample dilution (*x* axis) [30].

### 2.5. Pathogenicity Tests

#### 2.5.1. Schedule of Challenge

Five QX-type IBVs, GH/15, DYW/16, MS/17, DYYJ/17, and PZ/17, were chosen to infect SPF chickens. A total of 122 ten-day-old SPF chickens were randomly divided into six groups (A–E, *n* = 22 chickens each; F, *n* = 12 chickens). The birds of Groups A–E were inoculated with 100 μL of five QX-type strains (GH/15, DYW/16, MS/17, DYYJ/17, and PZ/17) containing 10^5^EID_50_, via intraocular and intranasal routes. The birds of Group F were inoculated with sterilized PBS as control. Chickens were held in separate biosafety level 2 (BSL2) isolators in the Laboratory Animal Center of Sichuan Agricultural University (Ya’an, Sichuan, China) with ad libitum access to feed and water; and they were maintained under uniform standard management conditions. The challenge schedule is shown in Table S2.

#### 2.5.2. Clinical Signs and Tissue Lesion

Ten chickens in Groups A–E and six chickens in Group F were used for the observation of clinical signs and tissue lesion. In detail, IB clinical symptoms, including sneezing, tracheal rales, chills, and increased drinking, were observed and recorded daily, until 8 days post-challenge (DPC). The morbidity and mortality of birds were calculated at the end of the study period.

**Figure 1:**
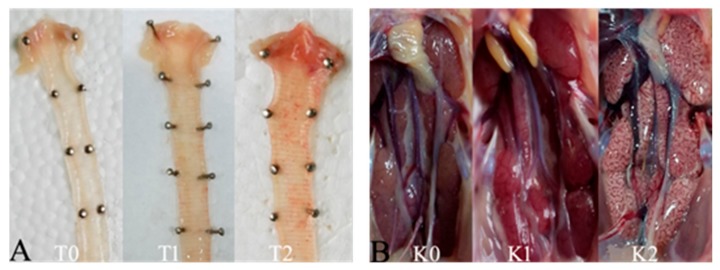
Gross lesion in trachea and kidney were observed. (**A**) The score standard in the trachea according to the extent: T0 (0 points) for normal, T1 (1 point) for abundant mucus and minor bleeders in the trachea, and T2 (2 points) for large hemorrhage in trachea. (**B**) The score standard in the kidney according to the extent: K0 (0 points) for normal, K1 (1 point) for kidney swelling, and K2 (2 points) for mottled kidney.

These chickens that died during the experimental period in each group were necropsied immediately, and the survival birds in each group were euthanized and necropsied at 8 DPC. Samples of the trachea and kidney were collected and saved in 10% neutral formalin for 24 h, embedded in paraffin, and stained with hematoxylin and eosin (HE) before being observed under a standard light microscope. Gross lesions of trachea were scored as follows: 0 points, normal; 1 point, abundant mucus and minor bleeders in the trachea; and 2 points, large hemorrhage in the trachea (Figure 1). Gross lesions of the kidney were scored as follows: 0 points, normal; 1 point, kidney swelling; and 2 points, mottled kidney (Figure 1). The mean lesion scores (MLS) in the trachea and kidney were calculated for each group.

#### 2.5.3. Virus Load Quantification

The twelve chickens in Groups A–E and six chickens in Group F were used for the quantification of virus shedding; four chickens were randomly selected for sampling the trachea and kidney at 4, 6, and 8 DPC. The samples of trachea and kidney were collected and processed as follows: after flushing the tracheal mucosa with 3 mL of PBS and grinding the kidney with 2 mL PBS, the fluids were collected and stored at −70 °C for further analysis. After freezing, thawing, and centrifugation at 1000× *g* for 30 min at 4 °C, three times, 200 μL of the supernatant was used for RNA extraction. Virus copies were determined by RT-qPCR, as described above.

### 2.6. Statistical Analysis

Data analysis of survival, lesion scores, and RT-qPCR were performed by using IBM SPSS Statistics 22 software. Statistical significance was considered as follows: probability (*p*) value < 0.05 was considered statistically significant.

### 2.7. Ethics Statement

All animal experiments were conducted in compliance with protocols approved by the Sichuan provincial Laboratory Animal Management Committee (Permit Number: XYXK (Sichuan) 2014-187). The protocols for this experiment were performed according to the guidelines of the Ethics and Animal Welfare Committee (EAWC) of the Sichuan Agricultural University.

## 3. Results

### 3.1. Phylogenetic and Recombination Analysis of the S1 Gene

**Figure 2:**
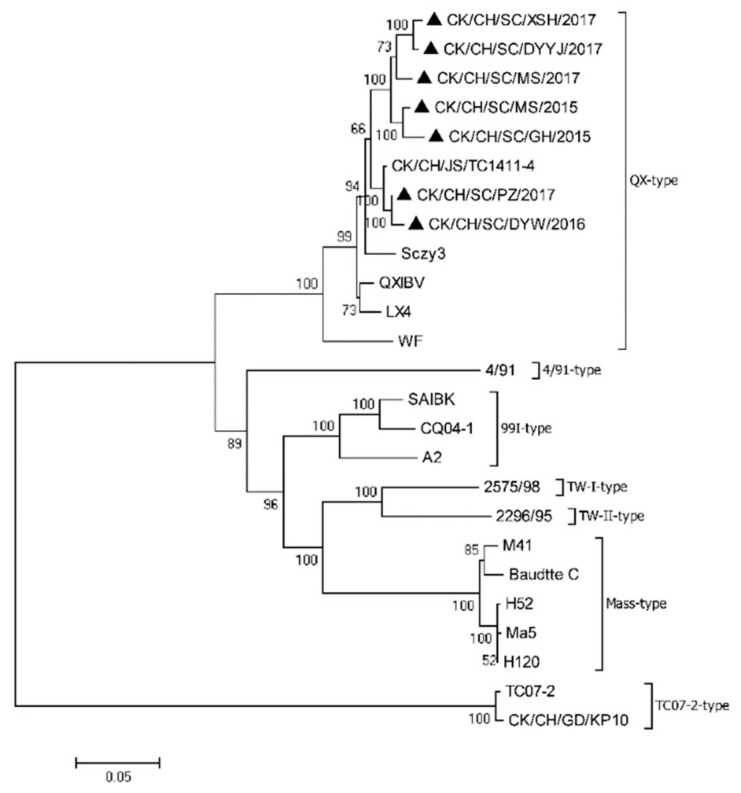
Phylogenetic tree was constructed based on the ORF nucleotide sequences of the S1 gene from seven field strains (filled triangles) and 18 reference strains, using MEGA version 7.0.14, with the neighbor-joining method and 1000 bootstrap replicates. The scale bar corresponds to 0.05 estimated amino acid substitutions per site.

Phylogenetic trees were constructed based on ORF nucleotide sequences of the S1 gene (Figure 2). All eight IBV field strains were QX genotype, sharing 94.3%–99.2% nucleotide identity of the S1 gene with each other and 90.6%–98.6% with other QX-type reference strains. The S1 gene identities of these two IBV isolates, DYW/16 and PZ/17, were highest 99.2%, while the lowest similarity was 94.3% between Sczy3 and XSH/17 isolates. Additionally, the nucleotide identity for the S1 gene was only 76.5%–78.7% between the eight IBV isolates and commercial vaccine strains (H120 and 4/91). Recombination analysis of the S1 gene sequence indicated no recombination event in the S1 genes of the seven field strains.

On analyzing the amino acid sequence, the S1 domain showed 92%–98.3% identity, and the hypervariable regions (HVR) were located at residues 23–65 and 120–393 among the eight isolates. Amino acid substitution between S (Ser) residues and other amino acid residues was frequently observed, with the highest substitution rate occurring in S and N (Asn). Interestingly, the S1 protein of DYW/16 had six amino acid differences at the sites 150 D (Aspartic acid), 151N (Asparagine), 163W (Tryptophan), 193I (Isoleucine), 240K (Lysine), and 241C (Cystine), compared with that of the other six field strains and Sczy3, H120, and 4/91.

### 3.2. Antigenic Analysis of the QX-Type Strains

In terms of the neutralization titer of the eight QX-type IBVs, the immune sera of all strains could neutralize each other, but the neutralization ability varied with different IBV strains. Immune sera of DYYJ/17 showed high neutralizing ability against MS/17, XSH/17, DYYJ/17, GH/15, PZ/17, Sczy3, and DYW/16 strains. Sczy3 and DYW/16 antisera showed high neutralizing ability against at least four strains. However, antisera of PZ/17 and GH/15 showed low neutralizing ability against other isolates; their mean neutralization titer was significantly lower than that of other antisera (Table 2).

**Figure 3:**
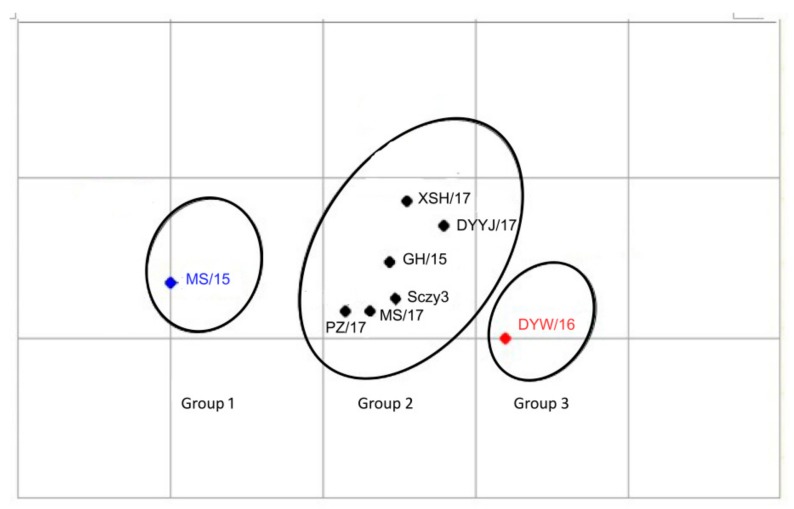
Map of the 8 QX-type IBV isolates. Antigenic cartography representations of the neutralization titer generated by using chicken antisera. Viruses in the same group were supported with the same color.

The seven QX-type isolates were classified into the same serotype as the Sczy3 strain, a representative strain of the SCZY3-serotype identified as the predominant serotype in Southwestern China during 2012–2016 [12,25]. Therefore, seven QX-type isolates could be clustered into the SCZY3-serotype, but the XSH/17 strain showed a loose relatedness with Sczy3 (0.11 ≤ R < 0.32) (Table 3). Of these seven IBV isolates, MS/15 and GH/15 showed a loose relatedness with DYW/16 (0.11 ≤ R < 0.32) (Table 3). AntigenMap analysis showed that eight QX-type strains (including Sczy3) could be classified into three antigenic subgroups: six IBVs including the five IBV field isolates MS/17, XSH/17, DYYJ/17, GH/15, and PZ/17 and Sczy3 strain were grouped into subgroup 2, while MS/15 while MS/15 strainwas placed in subgroup 1 and DYW/16 strain belonged to subgroup 3 (Figure 3).

### 3.3. Establishment of a Standard Curve

Standard curves were plotted with the log of target gene copy numbers as the *x* axis and cycle threshold (Ct value) as the *y* axis. The efficiency of the standard curves was 94%, the correlation coefficient (R2) of the standard curve was 1.0000, and the concluding equation of the standard curve was Y = − 3.659lgX + 4.16 (*y* axis is the threshold cycle (Ct); *x* axis is the initial copy number present in each sample). To verify the specificity of a particular primer, a dissolution curve was generated at the end of the cycle, and the result showed only the target product as a single melt peak.

### 3.4. Pathogenicity Tests

#### 3.4.1. Clinical Signs

**Figure 4:**
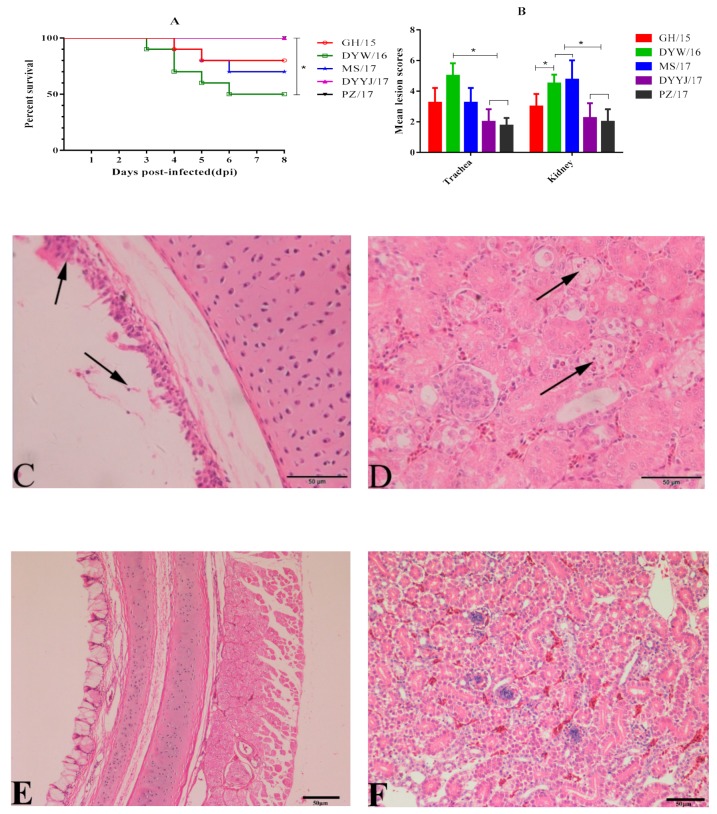
(**A**): Survival percentage of ten chickens per group after challenge IBV (statistical differences between different groups were determined by using the analysis of Log Rank test. * *p* < 0.05 indicates a significant difference). (**B**) Trachea and kidney MLS of ten chickens per group infected with IBV (statistical differences between different groups were determined by using the analysis of Mann–Whitney U test. * *p* < 0.05 indicates a significant difference). (**C**) Degeneration and necrosis of the ciliated epithelial cells (black arrow). (**D**) Degeneration and necrosis in the renal tubules kidney (black arrow). E: normal trachea. F: normal kidney.

At 2–3 DPC, infected chickens in all groups began to display clinical symptoms such as sneezing, listlessness, and huddling. Different morbidity rates were observed in different groups: DYW/16, GH/15, MS/17, and DYYJ/17 showed 100% morbidity, and PZ/17 showed 70% morbidity. Dead chickens were observed in DYW/16, GH/15, and MS/17 groups at 3–6 DPC, whereas no dead chickens were observed in DYYJ/17 and PZ/17 groups (Figure 4A).The percentage survival of DYW/16isolates was 50% during the eight-day observation period. Significant differences were analyzed between DYW/16 group and group of DYYJ/17 and PZ/17 (*p* < 0.05). Survival rates of MS/17 and GH/15 isolates were 70% and 80%, respectively. There were no clinical symptoms or deaths observed in the control group birds.

#### 3.4.2. Gross and Microscopic Lesions

For routine gross anatomy in challenge groups, obvious pathogenic differences in the trachea and kidney were observed in five QX-type fields groups, while no gross lesion was observed in the control group. However, different degrees of gross lesions were clearly observed in each group by calculating MLS (Figure 4B). The trachea MLS in groups DYW/16, GH/15, and MS/17 was not significantly different. The DYW/16 group showed trachea MLS of 5 was highest, and the kidney MLS of 4.5 was higher than groups GH/15, PZ/17, and DYYJ/17 (*p* < 0.05). The trachea MLS of 3.25 was similar in MS/17 and GH/15 groups and higher than groups PZ/17 and DYYJ/17, but kidney MLS of 4.75 in group MS/17 strain was highest. For PZ/17 and DYYJ/17 groups, trachea and kidney MLS both were below 3 and lower than other groups.

The microscopic lesions in the trachea and kidney collected from each of the challenged chickens were further characterized. Except for the control group, microscopic lesions were both observed the groups DYW/16, MS/17, GH/15, PZ/17, and DYYJ/17, which were typically characterized as degeneration and necrosis of the ciliated epithelial cells (Figure 4C), as well as degeneration and necrosis in the renal tubules of the kidney (Figure 4D). The difference in trends of microscopic lesions between the five groups was consistent with the result of gross lesions observation in each group.

#### 3.4.3. Viral Load in the Trachea and Kidneys

**Figure 5:**
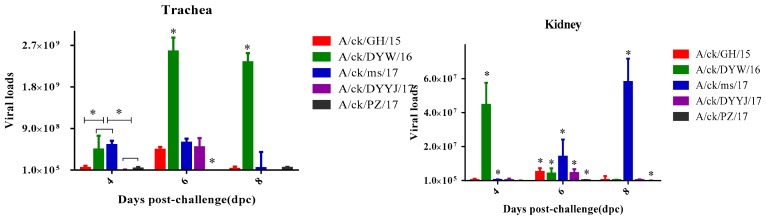
Virus loads of trachea and kidney in 12 chickens per group after challenge IBV were measured by RT-qPCR. Statistical differences between different groups were determined by using the analysis of one-way ANOVA method. * *p* < 0.05 indicates a significant difference.

The viral load in the trachea and kidneys from the infected groups are shown in Figure 5. For the DYW/16 strain, the tracheal viral load peak was observed at six DPC, and the kidney viral load peak was observed at four DPC. The viral loads of the DYW/16 strain in the trachea were significantly higher than other strains at 6–8 DPC (*p* < 0.05), and the viral loads in the kidney was significantly higher than other strains at four DCP. For the MS/17 strain, the peak of tracheal viral loads was six DCP, but the most virus loads of kidneys at eight DCP. The viral loads of the MS/17 strain were higher than other strains in infected chicken kidneys at 6–8 DCP (*p*< 0.05). For the GH/15, PZ/17, and DYYJ/17 strains, the viral loads in the trachea and kidney peaked at six DCP. In terms of total viral loads in the trachea and kidney, the DYW/16 strain showed higher values than other strains in the trachea, and the MS/17 strain showed higher values than other strains in the kidney. The viral loads of the DYYJ/17 in the trachea strain were lower than that of other strains, and the viral loads of the PZ/17 strain in the kidney were the lowest during the experiment period.

## 4. Discussion

IB is an economically important, highly contagious, acute, upper-respiratory tract disease of chickens and other fowl, caused by the avian coronavirus IBV [31,32]. Although multiple genotypes of IBV have continuously been detected, e.g., QX-type, TW-type, and TC07-2, QX-type IBV is still the prevalent genotype in China [12,15,33]. Therefore, it is important to understand the antigenicity and pathogenicity of QX-type IBV field isolates for the prevention and control of IB. It has been reported that the antigenicity and virulence vary between different genotypes [12,20], and no systematic comparison among QX-type IBVs has been conducted. In our study, eight QX-type IBVs were isolated from H120 and 4/91 vaccinated chickens in China, and their antigenicity and pathogenicity were analyzed for understanding the epidemiology and evolution of IBVs.

The S1 phylogenetic analyses revealed that eight QX-type isolates shared 94.3%–99.2% nucleotide identity with each other and were distantly related to H120 and 4/91 strains with 76.5%–78.7% nucleotide identity. The cross-reactivity R values indicated that the seven QX-type isolates were classified into the same SCZY3-serotype, which was different from H120 and 4/91 (R < 0.11) [12,25]. It has been reported that some QX-type strains showed distant relationships with other representative strains of the classical IBV serotypes [20]. The results indicated that different QX-type isolates were important agents for IBV outbreaks in China, and a new vaccine strain should be used for the prevention and control of IB. In the present study, higher neutralization ability against all analyzed strains came from immune serum of field isolates DYYJ/17 and DYW/16, implying that these strains have the potential to be used for the development of vaccine candidates.

The program AntigenMaphas been shown to effectively detect antigenic variationevents, and it is useful in antigenic characterization, which was applied for influenza, human enteroviruses, noroviruses, and porcine epidemic diarrhea virus [28,34]. In our study, eight QX-type IBVs with 94.3%–99.2% nucleotide identity were classified into the same serotype, but showed a minor difference in antigenicity among those strains. AntigenMap analysis showed that subgroup 2, which includes the Sczy3 strain and field isolates MS/17, XSH/17, DYYJ/17, GH/15, and PZ/17 had tight antigenicity, which has been majorly isolated since 2017. Meanwhile, subgroup 1 (MS/15 isolate) and 3 (DYW/16 isolate) were separated in 2015 and 2016. These results indicated that there areantigenic variations in different IBV strains over time, andminor changes in the S1 gene maycause antigenic variation. For example, the two strains of DYW/16 and PZ/17 shared 99.2% nucleotide identity, but were classified into different subgroups. However, the data of QX-IBVs used in this study are limited, and this map will be expanded further when more data becomes available in the future. It has been reported that antigenic relatedness values were influenced by hypervariable regions (HVR) in the S1 subunit of IBV S protein [8,35]. Therefore, antigenic variations mapped by AntigenMap may be related to the nucleotide mutations in HVR, which were located at residues 23aa–65aa and 120aa–393aa in the S1 protein of the eight QX-type isolates. Furthermore, amino acid substitution between S (Ser) residues and other amino acids’ residues, such as N (Asn) and P (Pro) in HVR, were frequently observed among the seven field isolates, but whether this has an effect on antigenic variations is unclear.

As we all know, pathogenicity of strains of IBV is variable, depending on the strain, age at exposure, and route of inoculation. In the present study, five QX-type isolates showed different pathogenicity to ten-old-day SPF chickens. The DYW/16 strain caused a mortality rate of 50%, and the MS/17 and GH/15 strains were respectively associated with a mortality rate of 30% and 20%, whereas the DYYJ/17 and PZ/17 strains only caused clinical symptoms and tissue lesion. At necropsy, the DYW/16, MS/17, and GH/15 strains could cause more server lesions in tracheas and kidneys than the DYYJ/17 and PZ/17 strains. However, the S1 gene between five QX-type IBVs with different pathogenicity shared 95.2%–99.2% identical. Such as the S1 gene, similarity is 99.2% between the DYW/16 and PZ/17 strains, indicating some key nucleotide mutation in the IBV S1 gene can lead to difference in pathogenicity [21]. Previous studies have shown that the binding properties of S proteins have been hypothesized to be associated with differences in pathogenicity [36]. The receptor-binding domain (RBD) in the spike protein of the M41 strain overlapped with HVR in the S1 gene was critical for attachment in the spike protein of IBV, and Y43H change in RBD of recombinant ArkDPC S1 proteins allows for enhanced binding to the trachea [10,35]. HVRs of five QX-type field isolates were located at residues 23aa–65aa and 120aa–393aa, and the six unique amino acids (D150, N151, W163, I193, K240, and C241) in HVRs of the virulent DYW/16 strain were observed, which may perform an important role in the pathogenicity.

The pathogenicity of IBV is determined by several factors, which may involve replication efficacy, tissue tropism, and the ability to deal with the host’s immune system. In the present study, RT-qPCR examination and tissue lesions both showed that the elevated levels of viral replication efficacy and tissue lesions in the trachea and kidney are associated with increased pathogenicity of IBV strains.The mechanisms of tissue tropism characteristics in different IBVs were elucidated, nephropathogenicB1648 strain disseminated to internal organs via a cell-free and -associated viremia with KUL01 + monocyticcells as important carrier cells, resulting in serious kidney lesions, whereas respiratory M41 does not do this [37]. The mechanism of replication in monocytic cells might be recruited to cause kidney lesion and even high mortality in chickens infected with five QX-type IBVs. Besides, it has been reported that QX-type IBV caused greater innate immune responses and induced greater apoptosis in CEK than the M41 strain, which is also a major contributing factor to the pathological outcome [38]. Therefore, QX-type IBVs have a strong ability to combat the host immune system, causing tissue lesion and even death. However, the mechanism responsible for the differences in the combat abilityremains unclear for different QX-type IBVs. Although, the S gene, replicase gene, and 5a accessory gene have also been reported to be responsible for IBV virulence [11,39], this still cannot completely explain the mechanism behind the virulence differences of IBVs. The key gene and amino acid residues associated with IBV virulence and antigenicity should be verified via new methods, such as the reverse-genetics technique.

## 5. Conclusions

In summary, QX-type isolates were important agents for IBV outbreaks in China; their antigenic and pathogenic characteristics were analyzed in this study. The results showed that the eight QX-type IBV isolates belonged to the same serotype, but the antigenic variation over time was observed. Different pathogenicity, replication efficacy, and tissue lesions in 10-day-old SPF chickens were also observed among QX-type isolates. In addition, the eight strains were distantly associated with H120 and 4/91 vaccine strains with 76.5%–78.7% nucleotide identity, suggesting the need for a new vaccine strain to prevent and control IB.The immune serum of two IBVs, DYYJ/17 and DYW/16, displayed good neutralization activity; therefore they have the potential to be developed as candidate vaccine strains.

## Figures and Tables

**Table 1:** Information of seven infectious bronchitis virus (IBV) isolates.

Strain.	Year of Isolation	Chicken Type	Location	Symptoms Phenotype	Abbreviations
CK/CH/SC/GH/2015	2015	Broiler	Sichuan (Guanghan)	Respiratory	GH/15
CK/CH/SC/MS/2015	2015	Broiler	Sichuan (Chengdu)	Respiratory	MS/15
CK/CH/SC/DYW/2016	2016	Broiler	Sichuan (Deyang)	Respiratory; Nephritis	DYW/16
CK/CH/SC/XSH/2017	2017	Broiler	Sichuan (Deyang)	Respiratory; Nephritis	XSH/17
CK/CH/SC/PZ/2017	2017	Broiler	Sichuan (Penzhou)	Respiratory	PZ/17
CK/CH/SC/MS/2017	2017	Broiler	Sichuan (Meishan)	Respiratory; Nephritis	MS/17
CK/CH/SC/DYYJ/2017	2017	Broiler	Sichuan (Deyang)	Respiratory	DYYJ/17

**Table 2:** The neutralization titer in CEK cell.

Virus	Antiserum							
	Sczy3	MS/15	GH/15	DYW/16	XSH/17	MS/17	DYYJ/17	PZ/17
Sczy3	**1240**	178	178	1995	45	501	1412	178
MS/15	1240	**1412**	708	501	708	356	708	1000
GH/15	625	178	**178**	501	708	501	5623	89
DYW/16	625	89	32	**1000**	251	178	1412	89
XSH/17	1240	251	178	1995	**501**	708	2818	126
MS/17	870	708	126	251	356	**356**	1412	63
DYYJ/17	1240	708	45	2818	251	251	**2818**	126
PZ/17	1240	126	708	708	89	501	1000	**126**

Bold data means the VN end point titers of antisera against the homologous strain.

**Table 3:** The antigenicity correlation of eight IBVs.

Virus	Antiserum							
	Sczy3	MS/15	GH/15	DYW/16	XSH/17	MS/17	DYYJ/17	PZ/17
Sczy3	*1.00*	**0.38**	0.71	0.60	0.28	0.99	0.71	1.18
MS/15		*1.00*	0.71	0.17	0.50	0.71	0.35	0.84
GH/15			*1.00*	0.30	1.18	0.99	0.71	1.68
DYW/16				*1.00*	1.00	0.35	1.19	0.71
XSH/17					*1.00*	1.19	0.70	0.42
MS/17						*1.00*	0.60	0.84
DYYJ/17							*1.00*	0.59
PZ/17								*1.00*

Italics data means the antigenic relatedness values of the homologous IBV strain.

## Supplementary Materials

The following are available online at https://www.mdpi.com/1999-4915/11/12/1154/s1: Table S1: The information of 18 IBV reference; Table S2: The challenge schedule of 5 QX-genotype IBVs.
