# Supplementary material for: Antigenic and Pathogenic Characteristics of QX-Type Avian Infectious Bronchitis Virus Strains Isolated in Southwestern China
Source: Viruses. 2019 Dec 13;11(12):1154. doi: 10.3390/v11121154 (PMC6950461; doi:10.3390/v11121154)
Supplement: Supplementary file 1 [file viruses-11-01154-s001.pdf]

Table S1: The information of 18 IBV reference

Strain	Country	Genotype	Yearsof isolation	Accession number
H120	Vaccine Strain	Mass	Vaccine Strain	EU822341
H52	Vaccine Strain	Mass	Vaccine Strain	AF352315
M41	Vaccine Strain	Mass	Vaccine Strain	DQ834384
Beaudette	Vaccine Strain	Mass	Vaccine Strain	M95169
Ma5	Vaccine Strain	Mass	Vaccine Strain	AY561713
LX4	Xinjiang, China	LX4	1999	AY338732
QXIBV	Shandong ,China	LX4	1997	AF193423
ZY3	Sichuan, China	LX4	2009	JF732903
WF	Shandong.China	LX4	2016	DQ480151
CK/CH/JS/TC1411-4	Guangdong.China	LX4	2016	KX107820
4/91	Vaccine Strain	4/91	1992	AF093794
A2	Beijing, China	99I	1996	EU526388
SAIBK	Sichuan, China	99I	Unknown	DQ288927
CQ04-1	Chongqing,China	99I	2004	GQ265952
2296/95	Taiwan， China	TW-II	1995	AY606321
2575/98	Taiwan， China	TW-I	1998	AY606314
TC07-2	Guangdong.China	TC07-2	2016	GQ265948
CK/CH/GD/KP10	Guangdong.China	TC07-2	2012	HQ018919

Table S2: The challenge schedule of 5 QX-genotype IBVs

Group	Challenge (10^5 EID_{50})				Survival study and lesions observation (12-18 days old)	RT-qPCR assay(14,16,18 days old)
	Strain	Age (days old)	Numbers	Dose (ul)	Numbers	Numbers
A	CK/CH/SC/MS/17	10	22	100	10	12
B	CK/CH/SC/PZ/17	10	22	100	10	12
C	CK/CH/SC/DYYJ/17	10	22	100	10	12
D	CK/CH/SC/GH/15	10	22	100	10	12
E	CK/CH/SC/DYW/16	10	22	100	10	12
F	Sterilized PBS	10	12	100	6	6
